# Right ventricular volume overload reboots cardiomyocyte proliferation via immune responses

**DOI:** 10.1186/s12967-024-05839-8

**Published:** 2024-11-28

**Authors:** Chunxia Zhou, Yuqing Hu, Zhuoya Dong, Zheng Wang, Sixie Zheng, Debao Li, Yingying Xiao, Dian Chen, Hao Chen, Sijuan Sun, Lincai Ye, Haibo Zhang

**Affiliations:** 1 Department of Thoracic and Cardiovascular Surgery, Shanghai Children’s Medical Center, Shanghai Jiao Tong University School of Medicine, 1678 Dongfang Road, Shanghai, 200127 China; 2 Department of Cardiology, Shanghai Children’s Medical Center, Shanghai Jiao Tong University School of Medicine, Shanghai, China; 3 Department of Pediatric Intensive Care Unit, Ningbo Women and Children’s Hospital, Ningbo, Zhejiang China; 4 Department of Pediatric Surgery, Children’s Hospital of Fudan University, National Children’s Medical Center, Shanghai, China; 5 Department of Thoracic and Cardiovascular Surgery, Shanghai Children’s Hospital, School of Medicine, Shanghai Jiao Tong University, Shanghai, China; 6 Department of Pediatric Intensive Care Unit, Shanghai Children’s Medical Center, Shanghai Jiao Tong University School of Medicine, 1678 Dongfang Road, Shanghai, 200127 China; 7 Institute of Pediatric Translational Medicine, Shanghai Children’s Medical Center, Shanghai Jiaotong University School of Medicine, Shanghai, China; 8 Shanghai Institute for Pediatric Congenital Heart Disease, Shanghai Children’s Medical Center, Shanghai Jiaotong University School of Medicine, Shanghai, China

**Keywords:** Volume overload, Cardiomyocyte, Proliferation, Immune response, CHD

## Abstract

**Background:**

Right ventricular volume overload (RVVO) is one of the most important hemodynamic characteristics in children with congenital heart disease (CHD) and heart failure, and cardiomyocyte (CM) proliferation is one of the most vital factors for improving cardiac performance. However, whether and how RVVO reboots CM proliferation remains elusive.

**Methods and results:**

We first created a neonatal RVVO mouse model via abdominal aorta and inferior vena cava-fistula microsurgery at postnatal day 7 (P7), the edge of CM proliferation window. We subsequently performed bulk RNA-seq, single cell RNA-seq/flow cytometry, and immunofluorescence staining on the right ventricles (RV) of RVVO mice at P14/P21, defined as prepubertal stage, revealing that RVVO temporarily reboots prepubertal CM proliferation via immune responses.

**Conclusions:**

In considering the importance of RVVO and CM proliferation, this study may bring an opportunity to create a novel paradigm to treat pediatric CHDs or heart failure.

**Graphical Abstract:**

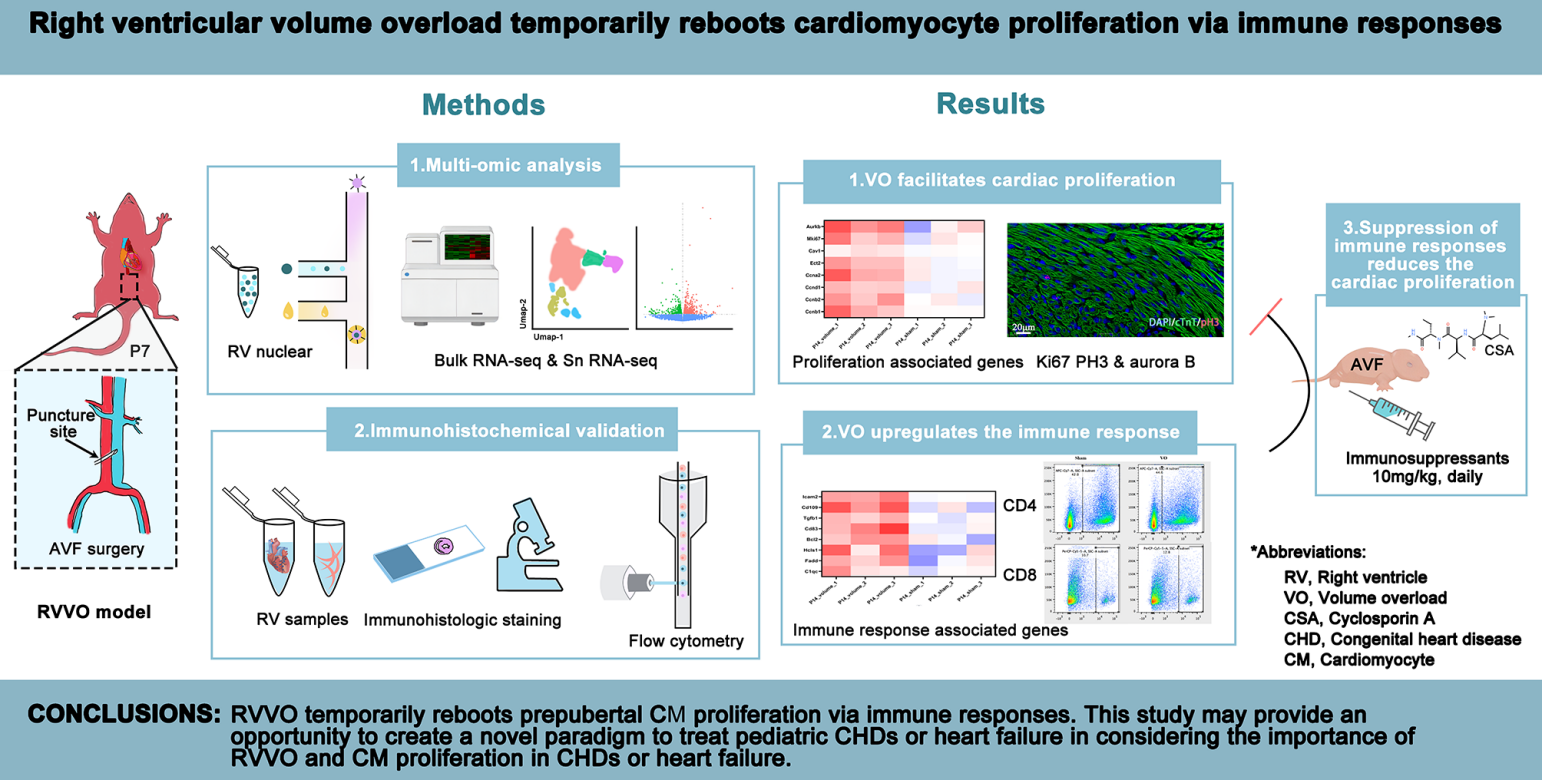

**Supplementary Information:**

The online version contains supplementary material available at 10.1186/s12967-024-05839-8.

## Introduction

Right ventricular volume overload (RVVO) is one of the most important hemodynamic conditions in many pediatric congenital heart diseases (CHDs), such as CHD with a left-to-right shunt (atrial septal defect, patent ductus arteriosus, and partial anomalous pulmonary vein drainage), CHD with right heart valve regurgitation (tricuspid regurgitation, pulmonary regurgitation, and repaired tetralogy of Fallot), and CHD with functional univentricular conditions (hypoplastic left heart syndrome and Fontan surgery) [1–7]. RVVO is also a hallmark of heart failure [8, 9]. Moreover, cardiomyocyte (CM) proliferation, the foundation of heart regeneration, contributes significantly to cardiac performance; thus, CM proliferation is an important consideration in CHD and heart failure treatment [10, 11]. However, most CMs lose proliferation ability soon after birth [12, 13]. For mice, the CM proliferation window is from postnatal day (P) 1 to P7 [12]. Therefore, understanding whether and how RVVO reboots CM proliferation may provide the foundation for a novel paradigm for treating pediatric CHD and heart failure.

Animal models of disease are critical in preclinical research; however, corresponding neonatal mouse/rat models for RVVO are lacking, limiting basic studies [1, 14]. How RVVO reshapes postnatal right ventricle (RV) development has largely been unknown due to the lack of a neonatal mouse/rat RVVO model [15–17]. The reason is that genetically defective neonatal mice/rats cannot be born or die immediately after birth [1, 14]. The only current approach is to construct a neonatal mouse/rat model of RVVO through microsurgical techniques [1, 14]. We created the first neonatal mouse/rat RVVO models via neonatal abdominal aorta and inferior vena cava fistula (ACF) microsurgery, which leads to a left-to-right shunt, at P1 or P7 [1, 15–17]. Using these models, we preliminarily demonstrated that RVVO initiates an immune response in the RV at the neonatal stage (from P1 to P7) and changes the postnatal RV developmental track (from P7 to P21) [1, 15–17].

Although there was an enrichment of cell cycle-related genes in the RVVO-dominant RV development track, it is unclear whether RVVO reactivates the proliferation of CMs [15, 16]. In this study, we performed ACF microsurgery at P7, the edge of CM proliferation window (P1–P7) and used bulk RNA-seq and single RNA-seq to reveal the role and potential mechanisms of RVVO in rebooting prepubertal CM proliferation.

## Materials and methods

The data generated in this study are available from the corresponding author upon reasonable request. The bulk RNA-seq data and single RNA-seq data have been deposited in the GEO database (https://www.ncbi.nlm.nih.gov/geo) under Accession No. GSE157396 and No. GSE255054, respectively.

### ACF microsurgery

**Fig. 1:**
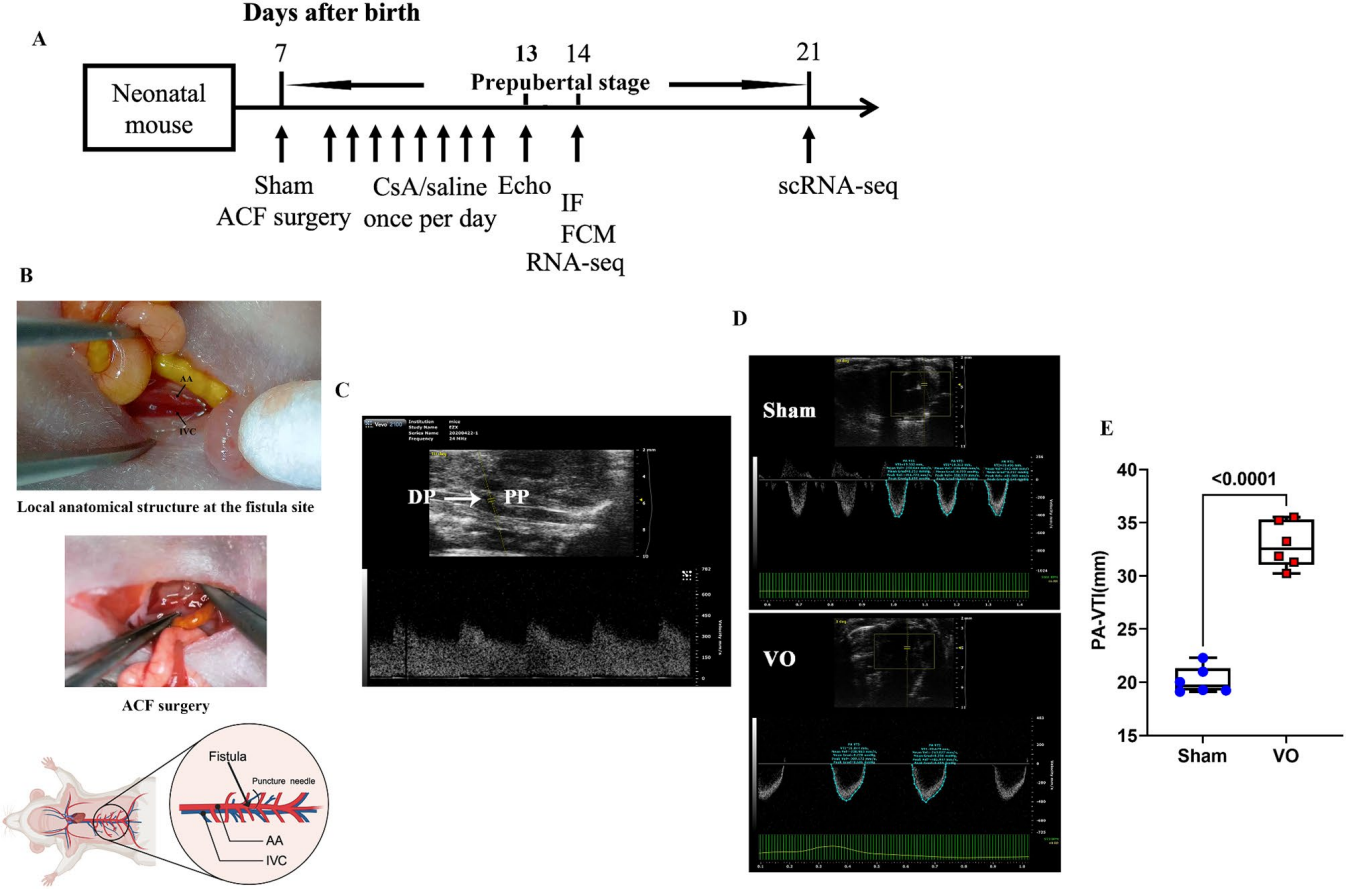
ACF microsurgery induces prepubertal RVVO. (**A**) Timeline of the experimental procedure. (**B**) Illustration of ACF microsurgery. (**C**) Wide and flat pulsatile blood flow was detected at the fistula; IVC: inferior vena cava; DP: detection point; AA, abdominal aorta; and PP: puncture point. (**D**) Representative image across the pulmonary artery valve. (**E**) Quantification of PA VTI

Pregnant C57BL/6 mice were purchased from Xipu’er-bikai Experimental Animal Co., Ltd. (Shanghai, China). At postnatal day 7 (P7), the neonate males or females were randomized into the RVVO and sham groups, which underwent the same procedure except for the puncture step. Neonatal ACF microsurgery was performed according to our previous publication [18]. In brief, P7 mice were anesthetized using 4% isoflurane, after which midline laparotomy was performed to expose the abdominal aorta (AA) and inferior vena cava (IVC). After puncturing through the AA into the IVC with an 11 − 0 nylon thread needle (Fig. 1A), hemostatic compression with the surrounding connective tissue was applied for 2 min. Finally, the abdominal wall was closed, and the pups were returned to their mother. The next day, the mice were injected subcutaneously on the back of the neck with cyclosporine A (12.5 mg/kg) or 0.9% saline for 6 days. All procedures conformed to the principles outlined in the Declaration of Helsinki and were approved by the Animal Welfare and Human Studies Committee at Shanghai Children’s Medical Center.

### Ultrasound

At P13, the mice were anesthetized with isoflurane (isoflurane/oxygen: 1.5–2.0% maintenance) for ultrasound examination. Fistula and pulmonary artery (PA) flow were analyzed with a Vevo 2100 imaging system (Visual Sonics, Toronto, ON, Canada). A long-axis view of the PA was used to measure the velocity time integral of the pulmonary artery (VTI_PA_, mm).

### Bulk RNA-seq analysis

At P14, defined as prepubertal stage [1], the RVs were extracted with the PureLink RNA Micro Scale Kit (12183016, Life Technologies, Carlsbad, CA, USA) to obtain total mRNA, which was subsequently used to generate sequencing libraries with the NEBNext^®^ UltraTM RNA Library Prep Kit (E7760, NEB, USA) following the manufacturer’s recommendations. The library was sequenced on the Illumina NovaSeq platform to generate raw data, which were processed through in-house PERL scripts to produce high-quality clean data. The clean data were subjected to differential expression analysis, Gene Ontology (GO), and Kyoto Encyclopedia of Genes and Genomes (KEGG) enrichment analyses. These analyses were performed on the Novo magic platform (https://magic.novogene.com/).

### Immunofluorescence staining

P14 hearts were sectioned and subjected to immunofluorescence staining. The slides were incubated with primary antibodies ((anti-sarcometric α actinin(SAA, Abcam, ab9465), anti-cardiac troponin T (cTnT, Abcam, ab8295), anti-phospho-histone H3 (pH3; Millipore, 06-570) and aurora B (Abcam, ab2254)) overnight at 4 °C. The slides were then incubated with secondary antibodies and 4’,6-diamidino-2-phenylindole (DAPI) for 30 min. ImageJ software (NIH, Bethesda, MD, USA) was used for quantification.

### Flow cytometry

At P14, the RVs were minced into small fragments and dissociated with 1:1 type II collagenase (1000 U/mL in PBS; Worthington, Lakewood, NJ, USA) and dispase (11 U/mL in PBS; Gibco, Waltham, MA, USA) at 37 °C for 30 min. The dissociated RV cells were incubated with red blood cell lysis buffer (eBiosciences, Waltham, MA, USA) for 5 min to remove erythrocytes. The cells were subsequently stained with fluorochrome-conjugated antibodies against CD45, CD4, and CD8 (BioLegend or eBiosciences) at a dilution of 1:100 at 4 °C for 30 min. After washing, the cells were analyzed on a flow cytometer (BD, FACSAria™ Fusion).

### Single-cell RNA sequencing and data analysis

At P21, defined as CM maturation stage [1], the RV tissues were harvested, minced, homogenized, and lysed in chilled Nuclei EZ Lysis Buffer. After washing with nuclei wash buffer, the nuclei were filtered through a 35-µm cell strainer. Then, the integrity and number of nuclei were determined by microscopy. Finally, the nuclei were processed using the standard 10X Genomics single-cell protocol [19]. The analyses were performed on the Shbio platform (http://tools.shbio.com/home/index.html) with the R package provided by Shanghai Biotechnology Corp. (Shanghai, China).

### Statistical analysis

Continuous data are expressed as the mean ± standard deviation. Differences were tested using Student’s *t* test if the data were normally distributed; otherwise, the rank sum test was used. P values < 0.05 were considered to indicate significance. Statistical analyses were performed using SAS software version 11.2 (SAS Institute, Inc., Cary, NC, USA).

## Results

### ACF microsurgery created RVVO in prepubertal mice

As shown in Fig. 1A–B, we performed ACF microsurgery on neonatal mice at P7, the edge of the CM proliferation window (P1–P7); then, we performed bulk RNA-seq, single RNA-seq, and associated examinations at P14 and P21 to investigate whether RVVO modulates CM proliferation. The inferior vena cava (IVC), which served as a negative control, had no pulsatile blood flow (Supplemental Fig. 1A); the abdominal aorta (AA), which served as a positive control, demonstrated pulsatile blood flow (Supplemental Fig. 1B). Additionally, there was broad and low pulsatile blood flow at the fistula (Fig. 1C) (Video 1), which induced RVVO, indicated by an increased velocity time integral (VTI) (Fig. 1D–E) (Video 2). These results were consistent with those presented in previous reports [15–18].

### RVVO reactivates prepubertal CM proliferation

P14 bulk RNA-seq revealed 981 differentially expressed genes (DEGs) between the VO and sham groups, 462 downregulated and 519 upregulated (Supplemental Fig. 2A). The heatmap and principal component analysis (PCA) results showed that the individual mice in the same group were similar but differed noticeably from the mice in the other group (Supplemental Fig. 2B–C). These results indicate that RVVO greatly changes the transcriptome of the prepubertal RV.

**Fig. 2:**
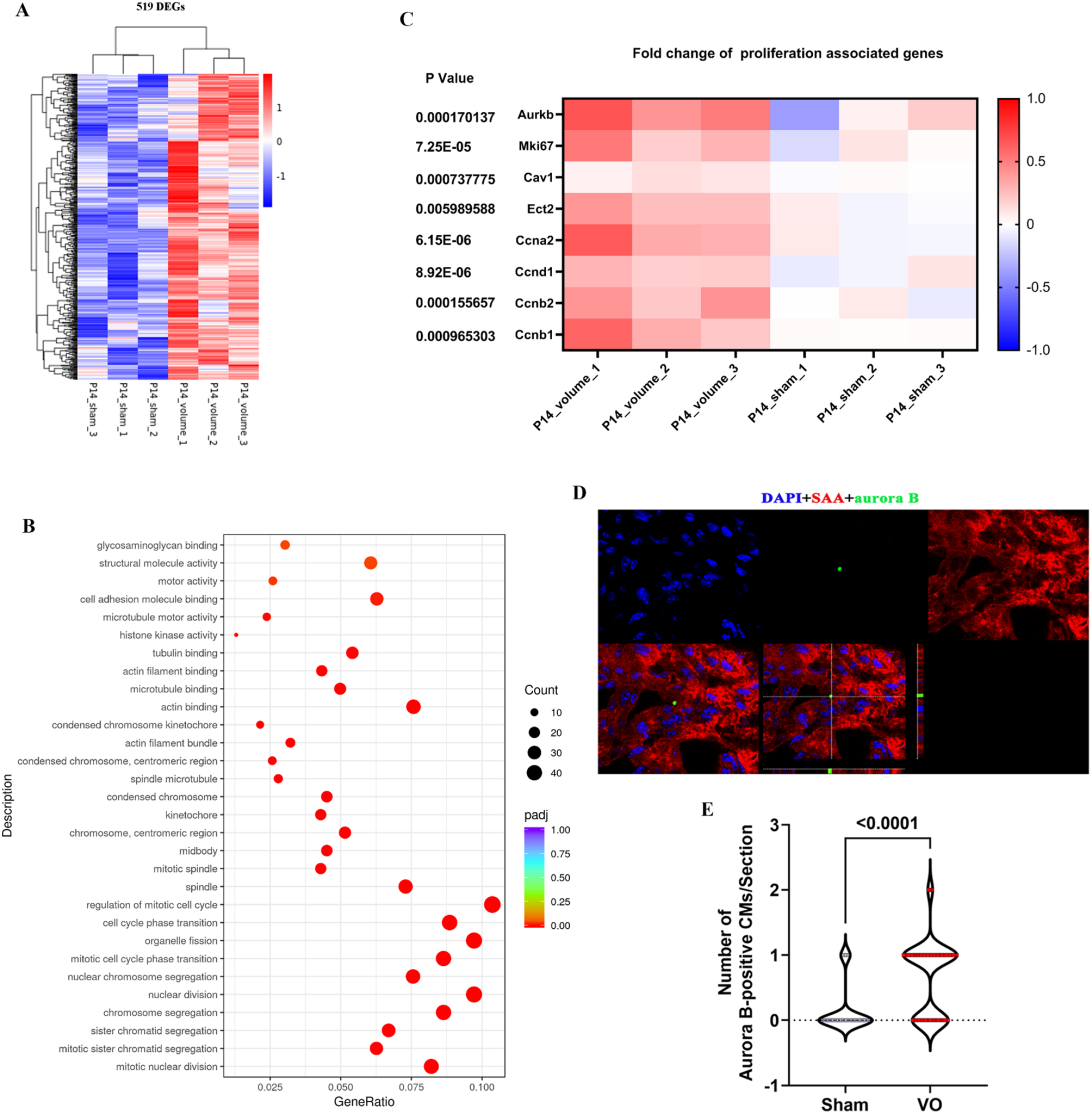
RVVO reboots prepubertal CM proliferation. (**A**) Heatmap of 519 upregulated DEGs. (**B**) Scatterplots of the top 30 enriched terms in the GO enrichment analysis of the upregulated DEGs. (**C**) Heatmap of the fold changes in the expression of cell proliferation-associated genes. (**D**) Representative aurora B-positive CM at the midbody. DAPI (blue); SAA(red); aurora B(green). (**E**) Quantification of aurora B-positive CMs in the midbody

**Fig. 3:**
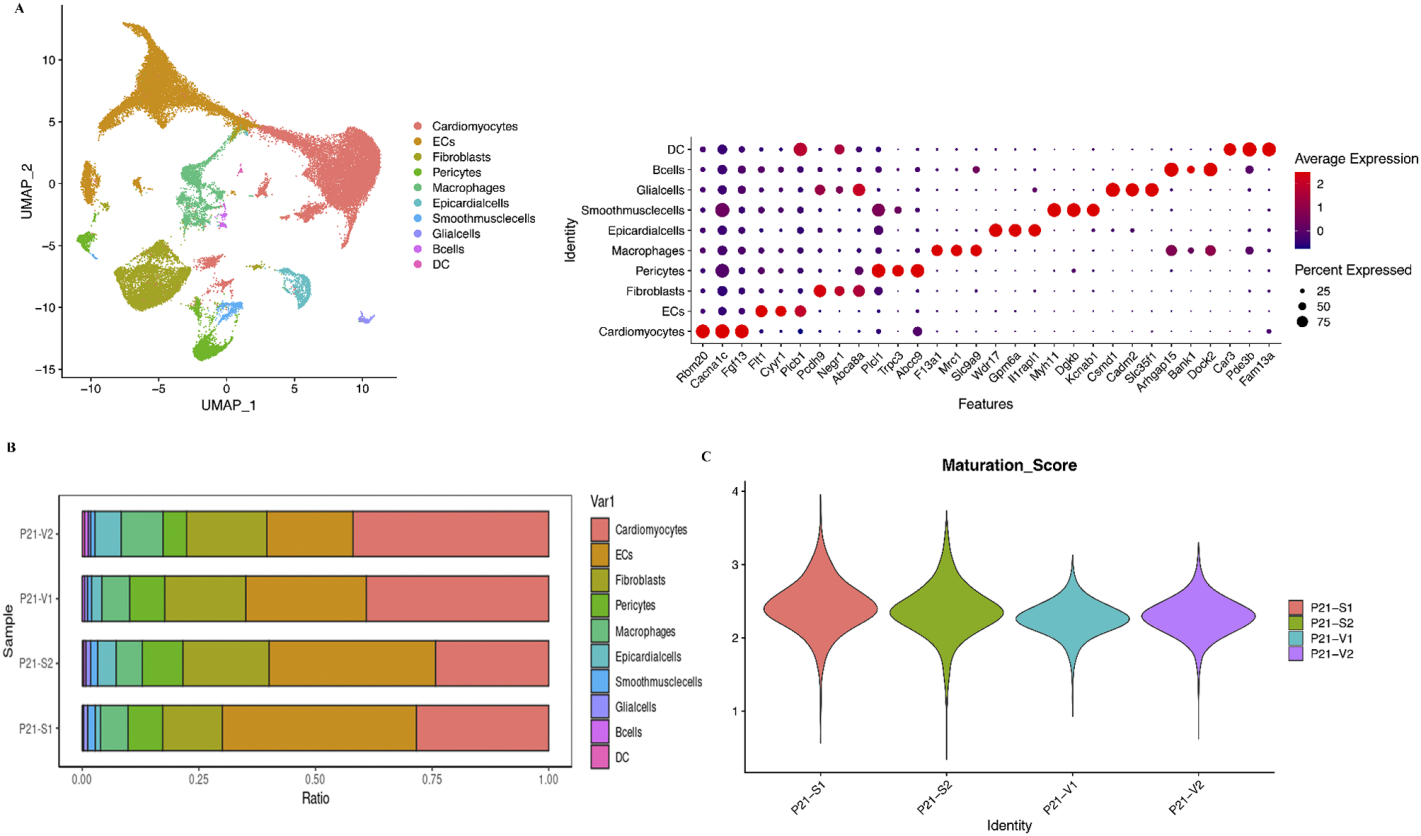
Single RNA-seq confirmed that RVVO reactivates prepubertal CM proliferation. (**A**) Left panel: UMAP plot of single RNA-seq data identifying 10 different types of cells; right panel: the expression levels of marker genes in each cell type. (**B**) Percentage of each cell type in each sample. (**C**) Maturation score of CMs in each sample

We subsequently subjected the upregulated and downregulated DEGs to GO and KEGG enrichment analyses (Fig. 2A). GO enrichment of the upregulated DEGs revealed that the top 30 enriched GO terms were associated with cell proliferation (Fig. 2B and Supplemental Fig. 3C). KEGG enrichment of the upregulated DEGs revealed that cell cycle was the fourth most enriched term (Fig. 3E). In addition, the heatmap of cell proliferation-associated genes showed that RVVO induced the upregulation of cytokinesis-associated genes (aurkb/ect2) (Fig. 2C). These results indicate that RVVO may reactivate prepubertal CM proliferation.

To confirm the findings revealed by RNA-seq, we assessed the mitosis marker pH3 and cytokinesis marker aurora B via immunofluorescence staining, which showed that RVVO significantly increased the number of pH3/aurora B-positive CMs (Supplemental Fig. 3A-B and Fig. 2D–E).

GO enrichment of the downregulated DEGs revealed that the top 30 enriched GO terms were associated with heart contraction (Supplemental Fig. 4A–C), suggesting that cardiac contraction ability was reduced because of RVVO. As there was a positive correlation between the increase in cardiac contractility and increase in CM maturation during postnatal cardiac development [20, 21], these results indicate that RVVO reduced CM maturation. In addition, CMs that can proliferate are considered immature CMs [22]. This result indirectly indicates that RVVO induces prepubertal CM proliferation.

We performed single RNA-seq on P21 RVs to confirm the above results. There were 10 different types of cells in the RVs (Fig. 3A). The percentage of CMs was significantly greater in the RVVO group than in the sham group (Fig. 3B). To further confirm these results, we calculated the maturation score for CM maturation genes (Myh6, Tnni3, RYR2, SERCA2, CACNA1C, and KCNJ2) [23] with Seurat’s functional module [24]. The results showed that the maturation score was lower in the RVVO group than in the sham group (Fig. 3C), suggesting that RVVO induces prepubertal CM proliferation.

### Immune responses account for prepubertal CM proliferation rebooting

**Fig. 4:**
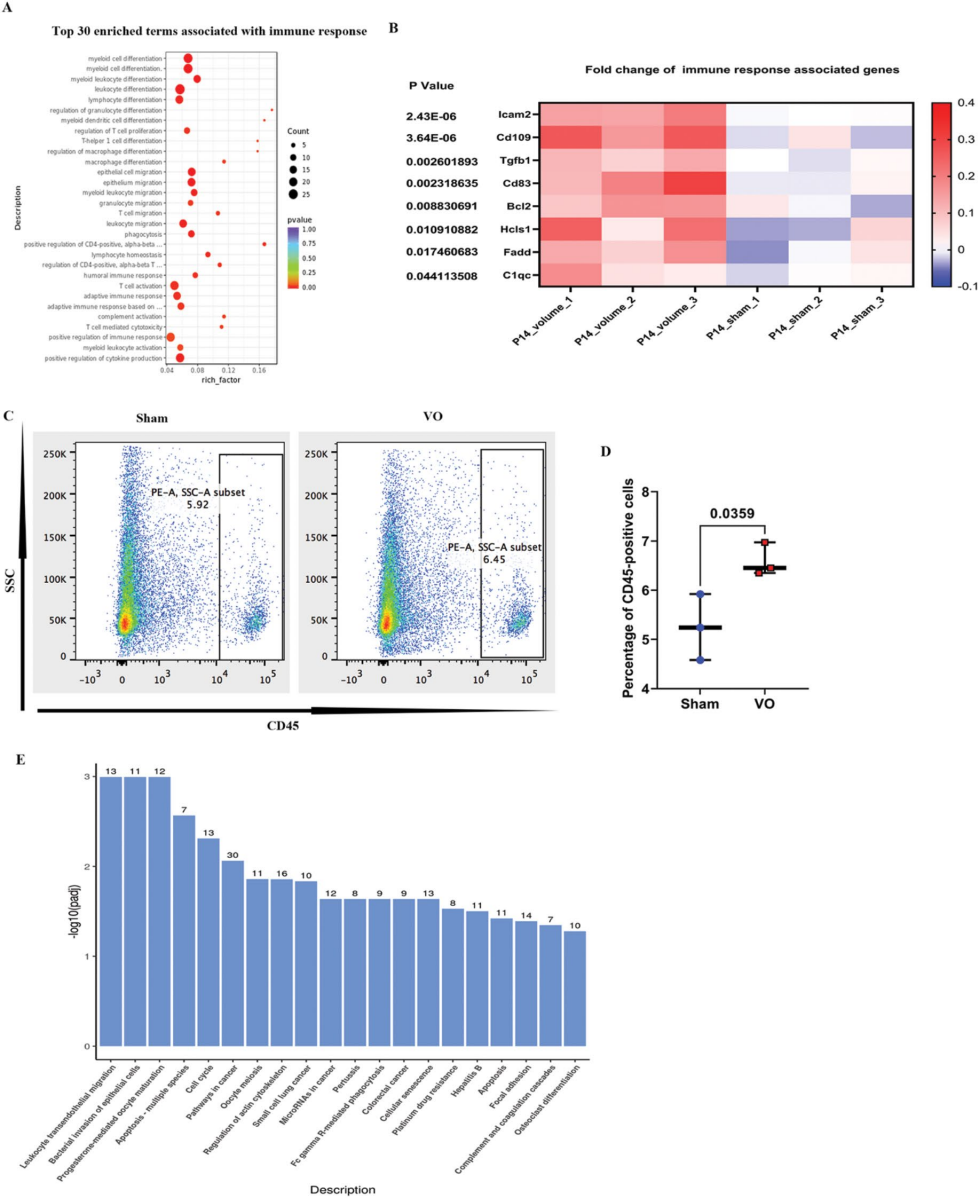
RVVO induces an immune response. (**A**) Scatterplots of the top 30 enriched GO terms associated with the immune response selected from GO enrichment analysis of the upregulated DEGs. (**B**) Heatmap of the fold changes in immune response-associated genes. (**C**). A representative flow cytometry image is shown. (**D**) Quantification of CD45 + cells. (**E**) The 20 most enriched terms in the KEGG pathway analysis of the upregulated DEGs

A previous study suggested that RVVO initiates immune responses at the neonatal stage (from P1 to P7) [17]; thus, we focused on the enriched GO terms of the upregulated DEGs. Immune response-associated terms were the second most enriched (Fig. 4A–B). We then assessed immune cells to confirm these results, revealing that RVVO increased the percentage of immune cells (Fig. 4C–D and Supplemental Fig. 5A–D). Moreover, immune response was one of the top three enriched KEGG pathway terms for the upregulated DEGs (Fig. 4E and Supplemental Fig. 5E). These results indicate that immune responses may account for prepubertal CM proliferation rebooting.

**Fig. 5:**
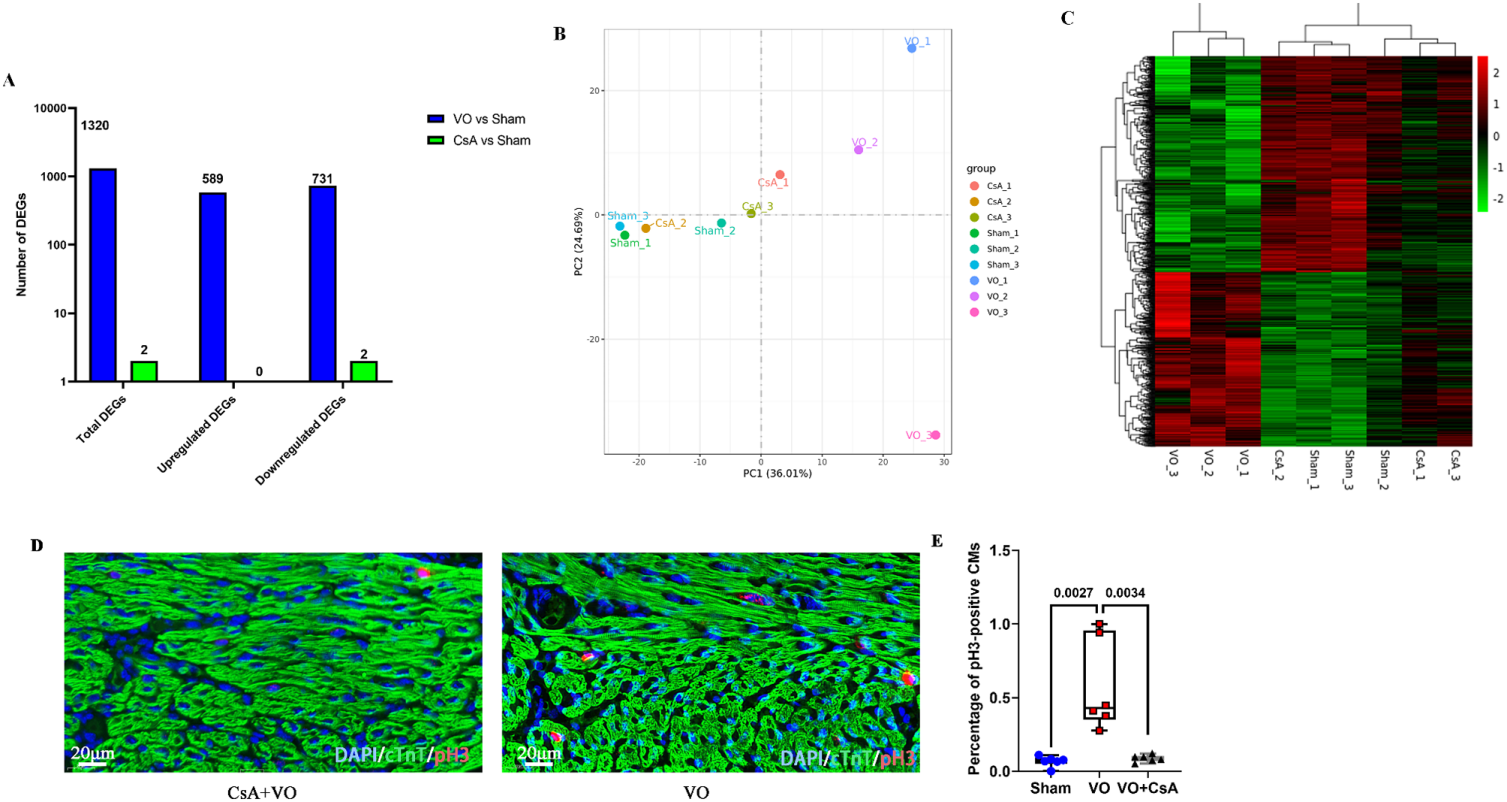
CsA inhibits prepubertal CM proliferation rebooting in RVVO (**A**) CsA reduced the number of DEGs. (**B**) CsA shifted the RVVO-changed gene expression profile toward the normal (sham) profile, as indicated by the PCA of the DEGs. (**C**) CsA shifted the RVVO-changed gene expression profile toward the normal (sham) profile, as indicated by the cluster analysis of the DEGs. (**D**) Representative immunofluorescence staining of pH3-positive CMs. (**E**) Quantification of pH3-positive CMs. Note: CsA = CsA + VO, CsA withou VO were comparable to the sham group

We treated mice with the immune response inhibitor cyclosporin A (CsA) to verify the above findings. CsA reduced the number of DEGs and caused the gene expression profile of RVVO mice to shift toward that of sham mice (Fig. 5A–C), with a significant decrease in cell cycle-associated gene enrichment (Supplemental Fig. 6). Moreover, CsA reduced the number of pH3-positive CMs induced by RVVO (Fig. 5D–E).

In summary, these results suggest that immune responses account for prepubertal CM proliferation rebooting.

## Discussion

Previous studies have shown that neonatal hearts can adapt well to abnormal hemodynamics in both the right and left ventricles without maladaptations such as fibrosis, partly due to CM proliferation [10, 25]. Even in early adolescence, CM proliferation can be induced by RVs to better adapt to pressure overload (PO) [11]. Our previous study also showed that relieving RV PO through debanding surgery at P7 significantly increased the survival rate of young mice [14]. All these studies suggest that the ability of pediatric hearts to adapt to abnormal hemodynamics, the main feature of CHD, is much greater than that of adult hearts.

CHDs usually lead to ventricular VO and PO, specifically RVVO, left ventricular VO, RV PO, and left ventricular PO, which usually coexist in the same type of CHD. Neglecting the unique hemodynamics of CHD when studying CM proliferation may lead to misinterpretation [14]. In addition, these hemodynamic abnormalities are closely associated with disease progression, and sometimes one type of hemodynamic abnormality dominates a particular stage of disease progression [26]. Previous studies have established that at the neonatal stage, PO promotes both RV and left ventricular CM proliferation [10, 25] and that at the prepubertal stage, PO induces transient RV CM cycle activity [11]. However, at the neonatal stage, RVVO does not promote CM proliferation but initiates an immune response [17]. The current study revealed that at the prepubertal stage, RVVO reboots CM proliferation and also induces an immune response, which may account for the RVVO-induced increase in CM proliferation. Thus, this study enriches the body of knowledge on CHD-associated CM proliferation. In addition, since RVVO is one of the most important hemodynamic characteristics in many kinds of pediatric CHD and heart failure, this study may provide an opportunity to create a novel paradigm for treating these diseases.

Current CHD treatment is limited by surgical approaches. Thus, finding non-surgical ways to treat CHD has always been an important goal of cardiac surgeons. When Liu et al. showed that beta-blockers improve CM proliferation via increasing the expression of ECT2 in children with tetralogy of Fallot (TOF), the most common cyanotic CHD and one that is characterized by RVPO, in the journal *Science Translational Medicine* [27] in late 2019, their results greatly interested cardiac surgeons [28–30] and quickly resulted in comments in *The New England Journal of Medicine* in early 2020 [31].

However, Liu’s argument is based on the increased polyploidy of CMs in children with TOF and the mutation of ECT2 gene leading to cytokinesis failure. However, the increased polyploidy of CMs may be the results of proliferation not cytokinesis failure (Supplemental Fig. S7). Moreover, subsequently, multiple studies revealed that PO promotes CM proliferation and the expression of ECT2 either in RV or left ventricle [10, 14, 25, 32, 33]. In all, Liu’s research overlooked the effect of PO on CM proliferation.

Why is CM proliferation potentially important in CHD treatment? In theory, CM proliferation leads to an increase in total CM number that may respond more favorably to both normal and abnormal stress and growth stimuli. In theory, it may set up a myocardial milieu more resistant to heart failure later in life. Thus, understanding the underlying mechanisms by which RVVO extends or reboots CM proliferation, it is possible that “we have at our disposal a simple, cheap medicine to treat a cellular-level problem that might have implications for long-term cardiac function.” [28].

CSA showed not only to improve RVVO-induced gene expression profile but also to inhibit reboot CM proliferation, and its broad immunosuppressive effects make it less than ideal for CHD treatment. Thus, it is necessary to investigate the exact immune cell types that play crucial roles in CM re-proliferation. Single-cell RNA sequencing results have suggested that macrophages are a potentially pivotal immune cell population. Specific types of immunosuppressants that target a corresponding immune cell population that will result in the fewest side effects should be identified. This will enable CHD patients to avoid a compromised immune state while still benefiting from agent administration.

### Limitations

The current study raises many interesting and important questions. For example, (1) whether the degree of RVVO is related to the degree of CM proliferation is unclear, and how to control the degree of RVVO is also unknown. (2) The specific mechanisms by which RVVO activates the immune response are unclear, and apoptosis or mechanical tension, which may be the underlying mechanisms by which RVVO activates the immune response, could be further researched. (3) It is unclear how immune responses reboot prepubertal CM proliferation. What are the key immune cells? Is it a macrophage? If so, how do macrophages reboot CM proliferation? According to our single-cell RNA-seq data, the interactions between CMs and macrophages increased dramatically (Supplemental Fig. 8). Thus, macrophages may be one of the underlying mechanisms responsible for VO-induced prepubertal CM proliferation. Answering the above questions will undoubtedly deepen our understanding of RVVO-associated CHD and heart regeneration.

## Conclusions

Given that RVVO is one of the most important hemodynamic characteristics in children with CHD and heart failure, the finding that RVVO reboots prepubertal CM proliferation may provide an opportunity to create a novel paradigm for treating these diseases.

## Electronic supplementary material

Below is the link to the electronic supplementary material.

Supplementary Material 1: Video 1. ACF microsurgery creates a fistula between AA and IVC.

Supplementary Material 2: Video 2. RVVO indicated by increased VTI.

Supplementary Material 3: Supplemental Figure 1 Abdominal ultrasound of the aorta and inferior vena cava. (A) No pulsatile blood flow in the inferior vena cava. (B) Pulsatile blood flow in the abdominal aorta (AA). Supplemental Figure 2 RNA-seq analysis of prepubertal RVs. (A) Volcano plot of differentially expressed genes (DEGs) between the RVVO and sham groups. (B) Heatmap of DEGs. (C) Principal component analysis (PCA) of DEGs. Supplemental Figure 3 RVVO reactivates prepubertal CM proliferation. (A) Representative immunofluorescence staining of pH3-positive CMs. DAPI (blue); SAA(green); pH3(red). (B) Quantification of pH3-positive CMs. (C) Histogram of the top 30 enriched terms in the GO enrichment analysis of the upregulated DEGs. Supplemental Figure 4 RVVO postpones prepubertal CM maturation. (A) Heatmap of the downregulated DEGs. (B) Histogram of the top 30 enriched terms in the GO enrichment analysis of the downregulated DEGs. (C) Scatterplots of the top 30 enriched terms in the GO enrichment analysis of the downregulated DEGs. Supplemental Figure 5 RVVO induces an immune response. (A) Representative flow cytometry image of CD4+ cells. (B) Quantification of CD4+ cells. (C) Representative flow cytometry image of CD8+ cells. (D) Quantification of CD8+ cells. (E) Scatterplots of the top 20 enriched terms in the KEGG pathway analysis of the upregulated DEGs.Supplemental Figure 6 CsA inhibits RVVO-mediated promotion of prepubertal CM proliferation. (A) Volcano plot of DEGs between the RVVO and sham groups. (B) Volcano plot of DEGs between the RVVO and CsA + VO groups. (C) Volcano plot of DEGs between the CsA + VO and sham groups. (D) Histogram of the top 30 enriched terms of GO enrichment analysis of the upregulated DEGs between the RVVO and sham groups. (E) Histogram of the top 30 enriched terms of GO enrichment analysis of the upregulated DEGs between the RVVO and CsA + VO groups. Supplemental Figure 7 Illustration of the increased polyploid CMs generated in TOF patients. Greater numbers of polyploid CMs do not indicate failure of cytokinesis. For example, in the beginning of a study, if both the control group and TOF group had 80 mononucleated and 20 binucleated CMs, the proportion of binucleated CMs was 20%. Under PO conditions, both mononucleated and binucleated CMs in the TOF group increased by 10, and the proportion of binucleated CMs was 25%. Therefore, an increase in the proportion of binucleated CMs does not necessarily mean impaired cytokinesis. (adopted from ref 1 under the CC BY license 4.0). Supplemental Figure 8 Number of interactions between macrophages and CMs. The thickness of the line represents the number of interactions.

## Data Availability

The data generated in this study are available from the corresponding author upon reasonable request. The bulk RNA-seq data and single RNA-seq data have been deposited in the GEO database (https://www.ncbi.nlm.nih.gov/geo) under Accession No. GSE157396 and No. GSE255054, respectively.

## Abbreviations

RVVO: Right ventricular volume overload
PO: Pressure overload
CM: Cardiomyocyte
RV: Right ventricle
ACF: Abdominal aorta and inferior vena cava fistula
