# Supplementary material for: Right ventricular volume overload reboots cardiomyocyte proliferation via immune responses
Source: J Transl Med. 2024 Nov 28;22:1075. doi: 10.1186/s12967-024-05839-8 (PMC11604012; doi:10.1186/s12967-024-05839-8)
Supplement: Supplementary file 3 — Supplementary Material 3: Supplemental Figure 1 Abdominal ultrasound of the aorta and inferior vena cava. (A) No pulsatile blood flow in the inferior vena cava. (B) Pulsatile blood flow in the abdominal aorta (AA). Supplemental Figure 2 RNA-seq analysis of prepubertal RVs. (A) Volcano plot of differentially expressed genes (DEGs) between the RVVO and sham groups. (B) Heatmap of DEGs. (C) Principal component analysis (PCA) of DEGs. Supplemental Figure 3 RVVO reactivates prepubertal CM proliferation. (A) Representative immunofluorescence staining of pH3-positive CMs. DAPI (blue); SAA(green); pH3(red). (B) Quantification of pH3-positive CMs. (C) Histogram of the top 30 enriched terms in the GO enrichment analysis of the upregulated DEGs. Supplemental Figure 4 RVVO postpones prepubertal CM maturation. (A) Heatmap of the downregulated DEGs. (B) Histogram of the top 30 enriched terms in the GO enrichment analysis of the downregulated DEGs. (C) Scatterplots of the top 30 enriched terms in the GO enrichment analysis of the downregulated DEGs. Supplemental Figure 5 RVVO induces an immune response. (A) Representative flow cytometry image of CD4+ cells. (B) Quantification of CD4+ cells. (C) Representative flow cytometry image of CD8+ cells. (D) Quantification of CD8+ cells. (E) Scatterplots of the top 20 enriched terms in the KEGG pathway analysis of the upregulated DEGs.Supplemental Figure 6 CsA inhibits RVVO-mediated promotion of prepubertal CM proliferation. (A) Volcano plot of DEGs between the RVVO and sham groups. (B) Volcano plot of DEGs between the RVVO and CsA + VO groups. (C) Volcano plot of DEGs between the CsA + VO and sham groups. (D) Histogram of the top 30 enriched terms of GO enrichment analysis of the upregulated DEGs between the RVVO and sham groups. (E) Histogram of the top 30 enriched terms of GO enrichment analysis of the upregulated DEGs between the RVVO and CsA + VO groups. Supplemental Figure 7 Illustration of the increased polyploid CMs generated in TOF [file 12967_2024_5839_MOESM3_ESM.pdf]

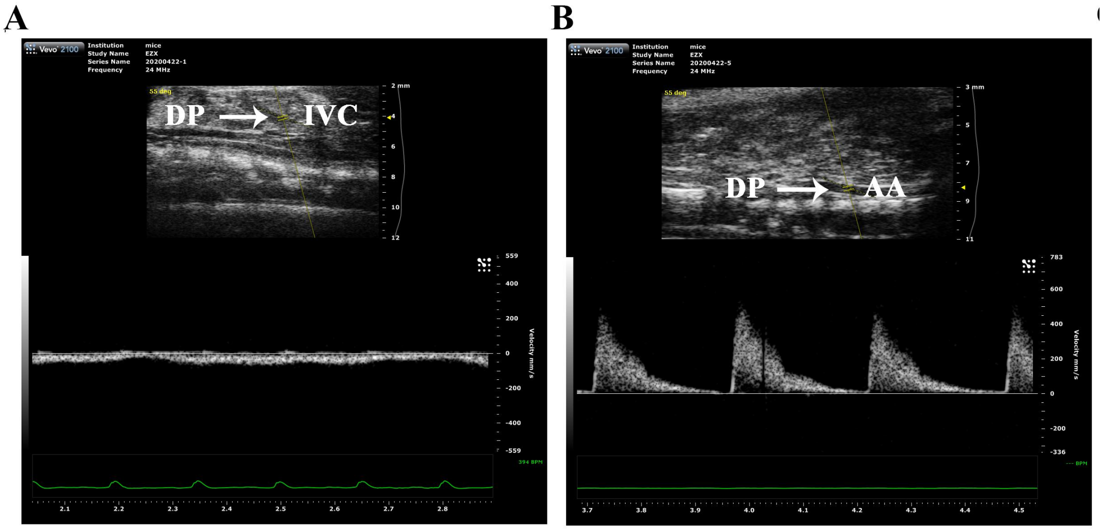

Supplemental Figure 1 Abdominal ultrasound of the aorta and inferior vena cava. (A) No pulsatile blood flow in the inferior vena cava. (B) Pulsatile blood flow in the abdominal aorta (AA).

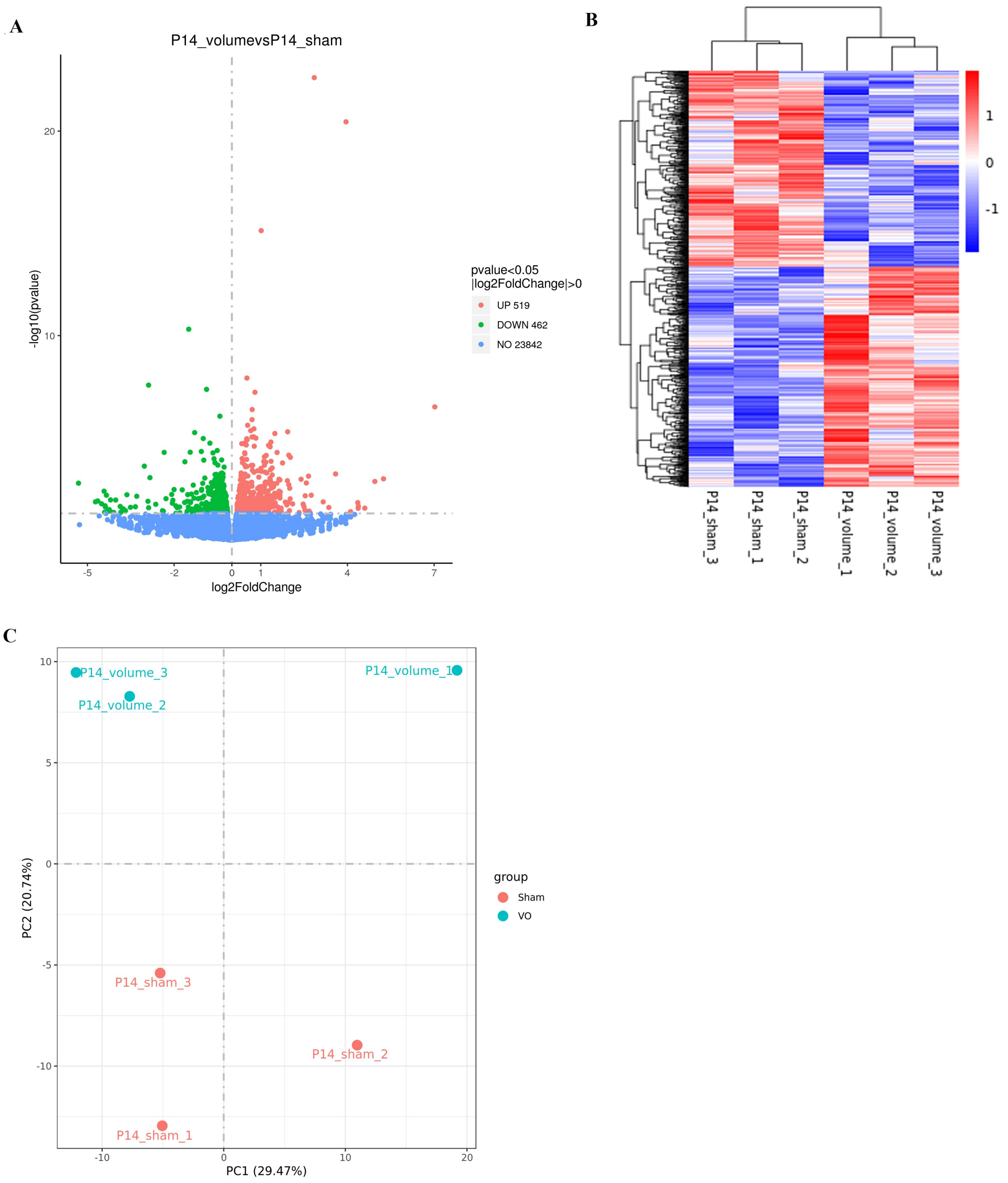

Supplemental Figure 2 RNA-seq analysis of prepubertal RVs. (A) Volcano plot of differentially expressed genes (DEGs) between the RVVO and sham groups. (B) Heatmap of DEGs. (C) Principal component analysis (PCA) of DEGs.

Supplemental Fig.S3

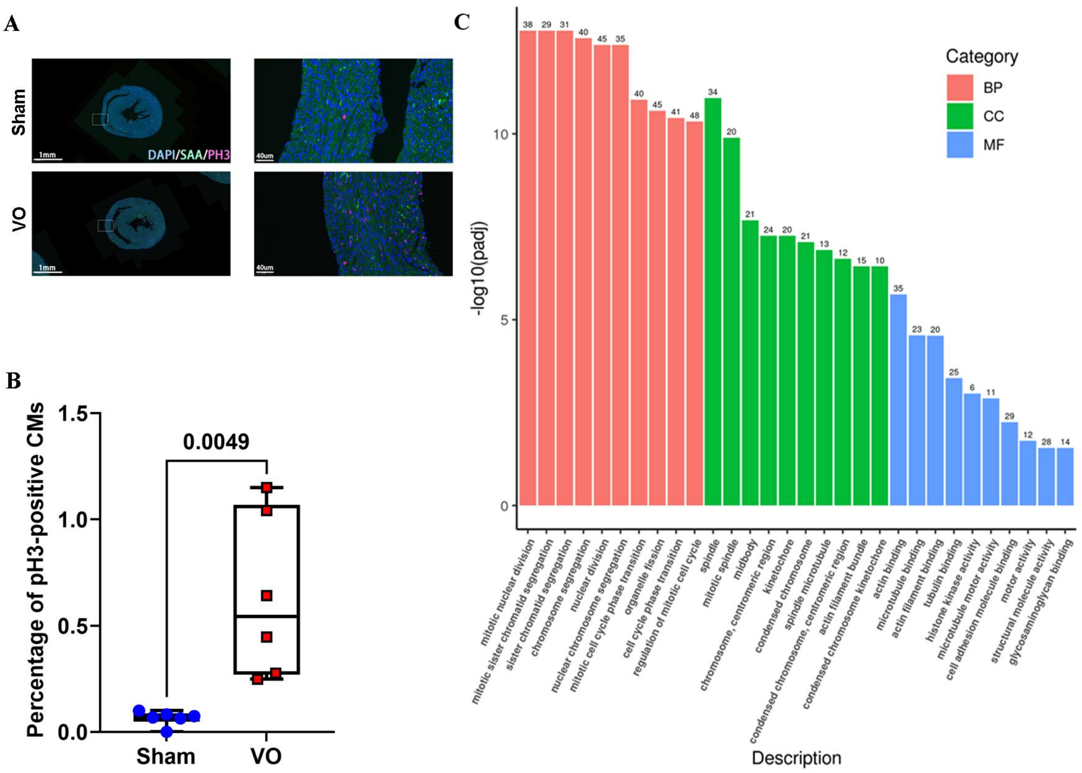

Supplemental Figure3 RVVO extends prepubertal CM proliferation. (A) Representative immunofluorescence staining of pH3-positive CMs.DAPI(blue); SAA(green); pH3(red). (B) Quantification of pH3-positive CMs. (C) Histogram of the top 30 enriched terms in the GO enrichment analysis of the upregulated DEGs.

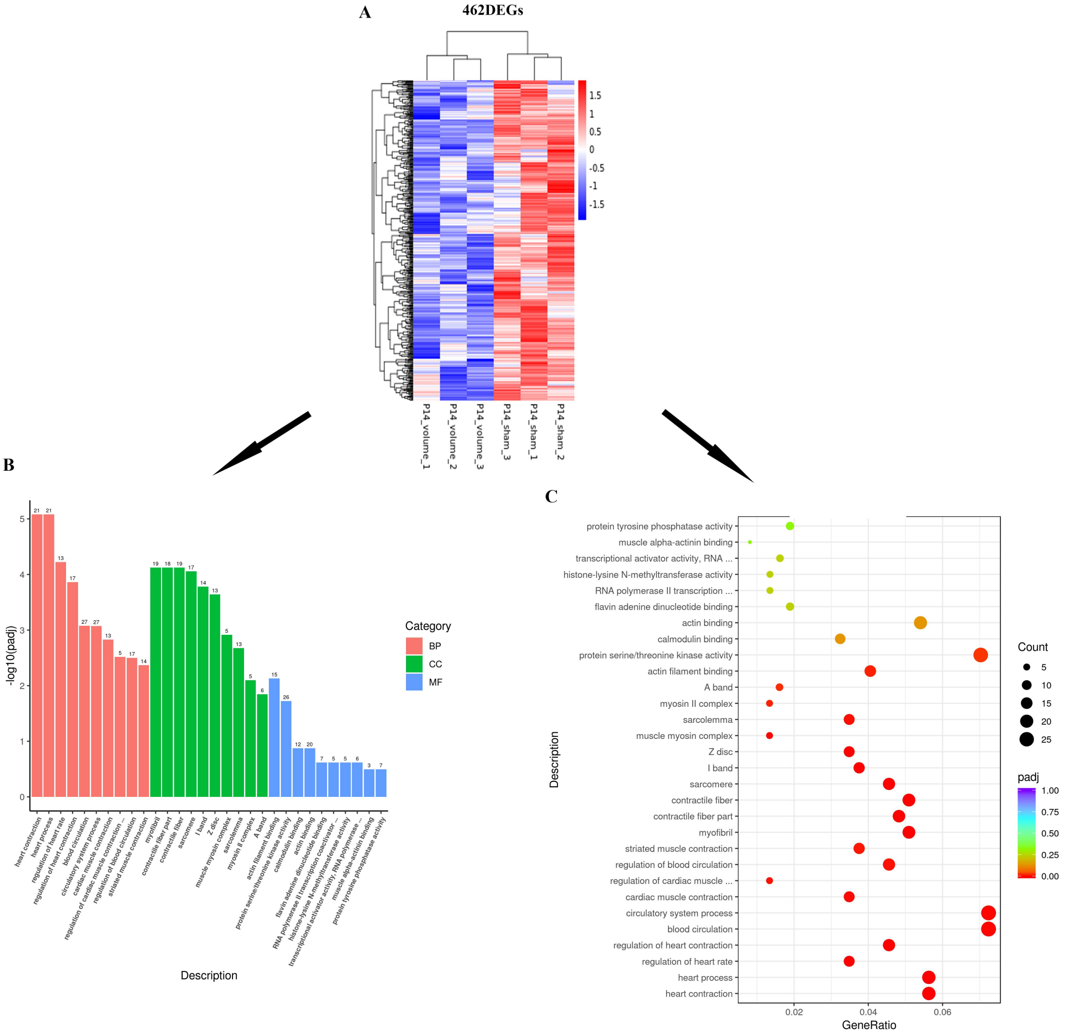

Supplemental Figure 4 RVVO postpones prepubertal CM maturation. (A) Heatmap of the downregulated DEGs. (B) Histogram of the top 30 enriched terms in the GO enrichment analysis of the downregulated DEGs. (C) Scatterplots of the top 30 enriched terms in the GO enrichment analysis of the downregulated DEGs.

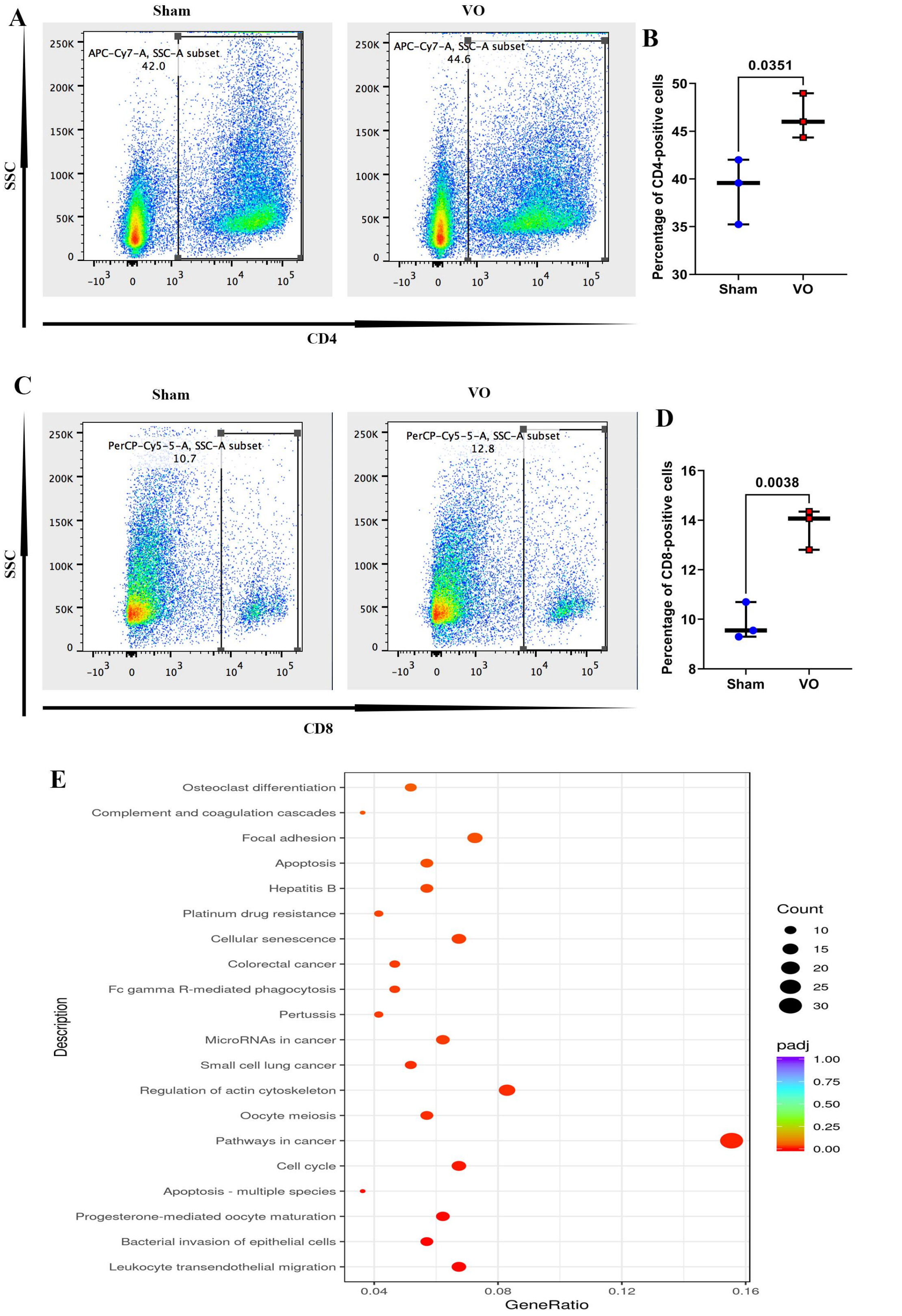

Supplemental Figure 5 RVVO induces an immune response. (A) Representative flow cytometry image of CD4+ cells. (B) Quantification of CD4+ cells. (C)

Representative flow cytometry image of CD8+ cells. (D) Quantification of CD8+ cells. (E) Scatterplots of the top 20 enriched terms in the KEGG pathway analysis of the upregulated DEGs.

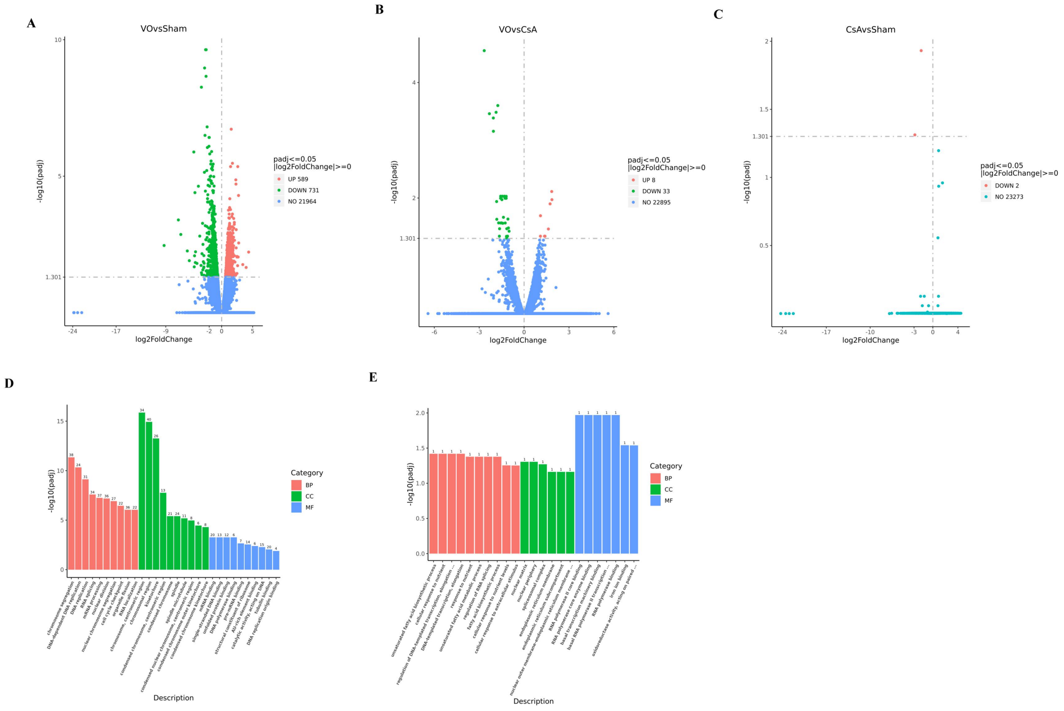

Supplemental Figure 6 CsA inhibits RVVO-mediated promotion of prepubertal CM proliferation. (A) Volcano plot of DEGs between the RVVO and sham groups. (B) Volcano plot of DEGs between the RVVO and CsA + VO groups. (C) Volcano plot of DEGs between the CsA + VO and sham groups. (D) Histogram of the top 30 enriched terms of GO enrichment analysis of the upregulated DEGs between the RVVO and sham groups. (E) Histogram of the top 30 enriched terms of GO enrichment analysis of the upregulated DEGs between the RVVO and CsA + VO groups.

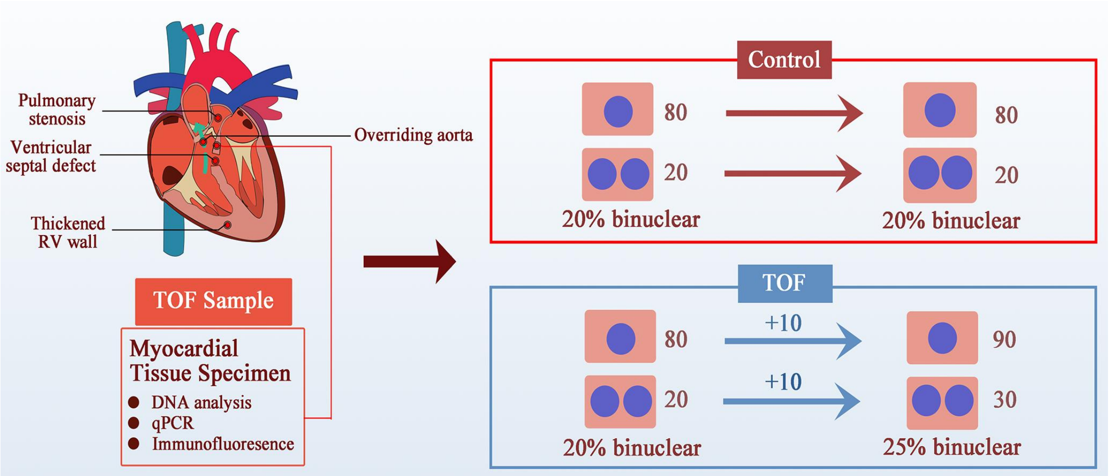

Supplemental Figure 7 Illustration of the increased polyploid CMs generated in TOF patients. Greater numbers of polyploid CMs do not indicate failure of cytokinesis. For example, in the beginning of a study, if both the control group and TOF group had 80 mononucleated and 20 binucleated CMs, the proportion of polyploid CMs was 20%. Under PO conditions, both mononucleated and binucleated CMs in the TOF group increased by 10, and the proportion of binucleated CMs was 25%. Therefore, an increase in the proportion of binucleated CMs does not necessarily mean impaired cytokinesis. (adopted from ref. 1 under the CC BY license 4.0)

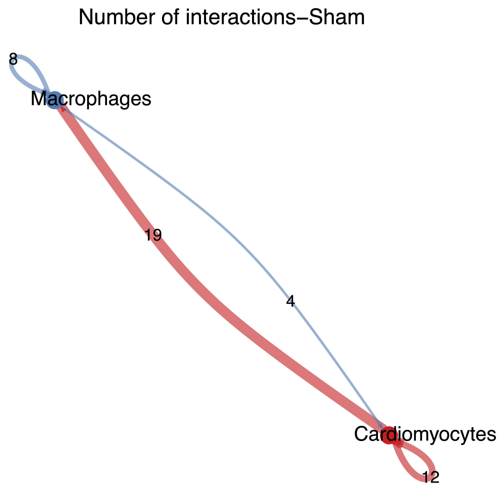

Supplemental Figure 8 Number of interactions between macrophages and CMs.

The thickness of the line represents the number of interactions.
